# Bamlanivimab therapy for acute COVID-19 does not blunt SARS-CoV-2–specific memory T cell responses

**DOI:** 10.1172/jci.insight.163471

**Published:** 2022-12-22

**Authors:** Sydney I. Ramirez, Alba Grifoni, Daniela Weiskopf, Urvi M. Parikh, Amy Heaps, Farhoud Faraji, Scott F. Sieg, Justin Ritz, Carlee Moser, Joseph J. Eron, Judith S. Currier, Paul Klekotka, Alessandro Sette, David A. Wohl, Eric S. Daar, Michael D. Hughes, Kara W. Chew, Davey M. Smith, Shane Crotty, for the Accelerating COVID-19 Therapeutic Interventions and Vaccines–2/A5401 (ACTIV-2/A5401) Study Team

**Affiliations:** 1 Center for Infectious Disease and Vaccine Research, La Jolla Institute for Immunology, La Jolla, California, USA.; 2 Division of Infectious Diseases and Global Public Health, Department of Medicine, University of California, San Diego, La Jolla, California, USA.; 3 Division of Infectious Diseases, Department of Medicine, University of Pittsburgh School of Medicine, Pittsburgh, Pennsylvania, USA.; 4 Department of Otolaryngology-Head and Neck Surgery, University of California, San Diego, La Jolla, California, USA.; 5 Division of Infectious Diseases and HIV Medicine, Department of Medicine, Case Western Reserve School of Medicine, Cleveland, Ohio, USA.; 6 Center for Biostatistics in AIDS Research, Harvard T.H. Chan School of Public Health, Boston, Massachusetts, USA.; 7 Department of Medicine, University of North Carolina at Chapel Hill School of Medicine, Chapel Hill, North Carolina, USA.; 8 Division of Infectious Diseases, Department of Medicine, David Geffen School of Medicine at University of California, Los Angeles, Los Angeles, California, USA.; 9 Eli Lilly and Company, San Diego, California, USA.; 10 Lundquist Institute at Harbor-UCLA Medical Center, Torrance, California, USA.; 11 See Supplemental Acknowledgments for ACTIV-2/A5401 Study Team member details.

**Keywords:** COVID-19, Immunology, Adaptive immunity

## Abstract

Despite the widespread use of SARS-CoV-2–specific monoclonal antibody (mAb) therapy for the treatment of acute COVID-19, the impact of this therapy on the development of SARS-CoV-2–specific T cell responses has been unknown, resulting in uncertainty as to whether anti–SARS-CoV-2 mAb administration may result in failure to generate immune memory. Alternatively, it has been suggested that SARS-CoV-2–specific mAb may enhance adaptive immunity to SARS-CoV-2 via a “vaccinal effect.” Bamlanivimab (Eli Lilly and Company) is a recombinant human IgG1 that was granted FDA emergency use authorization for the treatment of mild to moderate COVID-19 in those at high risk for progression to severe disease. Here, we compared SARS-CoV-2–specific CD4^+^ and CD8^+^ T cell responses of 95 individuals from the ACTIV-2/A5401 clinical trial 28 days after treatment with bamlanivimab versus placebo. SARS-CoV-2–specific T cell responses were evaluated using activation-induced marker assays in conjunction with intracellular cytokine staining. We demonstrate that most individuals with acute COVID-19 developed SARS-CoV-2–specific T cell responses. Overall, our findings suggest that the quantity and quality of SARS-CoV-2–specific T cell memory were not diminished in individuals who received bamlanivimab for acute COVID-19. Receipt of bamlanivimab during acute COVID-19 neither diminished nor enhanced SARS-CoV-2–specific cellular immunity.

## Introduction

In 2019, SARS-CoV-2 arose as a novel human pathogen and etiologic cause of COVID-19, a clinical syndrome ranging in disease severity from asymptomatic infection to severe pneumonia and death (1). Monoclonal antibodies (mAbs) have demonstrated efficacy against viral pathogens, like respiratory syncytial virus and Ebola (2), and were quickly developed and tested in clinical trials to identify safe and effective prophylaxis and treatments for COVID-19. The clinical efficacy and safety data from such trials resulted in emergency use authorization (EUA) from the US Food and Drug Administration (FDA) for several products and subsequent clinical use in adults and children at risk for severe COVID-19 (3, 4).

Although clinical trials have demonstrated the safety of SARS-CoV-2 neutralizing mAb (nAb) therapy and efficacy in preventing hospitalization and mortality from COVID-19, little is known regarding the effects of mAb therapy for acute COVID-19 on the development and maintenance of adaptive immunity to SARS-CoV-2 (5), in particular virus-specific CD4^+^ and CD8^+^ T cell responses. Because the early administration of SARS-CoV-2–specific mAb during acute COVID-19 can reduce viral burden and improve viral clearance (4, 6), there is concern that persons with acute infection who receive mAb therapy will develop inferior-quality adaptive immunity to SARS-CoV-2 due to reduced exposure to viral antigens (7). Alternatively, it has been hypothesized that mAb therapy could enhance antiviral adaptive immunity through a “vaccinal effect” (8–10).

NAbs are able to block viral entry into host cells, thereby preventing infection. When sufficient amounts of exogenous (e.g., mAb therapy) or endogenous (e.g., preformed from prior infection or immunization) nAbs are present, it is possible to achieve sterilizing immunity (11). Although in most persons with acute COVID-19 the initial, nonspecific, innate immune response is rapidly complemented by SARS-CoV-2–specific adaptive immune responses consisting of antibody-producing B cells (humoral immunity) and CD4^+^ and CD8^+^ T cells (cellular immunity), it takes time for this adaptive antiviral response to develop (11–13). During primary SARS-CoV-2 infection, many cells will become infected prior to the development of SARS-CoV-2–specific humoral immunity, and efficient viral clearance likely requires cellular immunity (11, 12, 14–17). Following acute infection and viral clearance, B and T cells ultimately provide long-lived protection in the form of memory B and T cells (13, 18–22). With T cell assistance, SARS-CoV-2–specific memory B cells (MBCs) undergo somatic hypermutation to form antibodies with increased affinity and neutralization potential to SARS-CoV-2 (22–24).

Most immunocompetent adults with primary SARS-CoV-2 infection develop SARS-CoV-2–specific CD4^+^ and CD8^+^ T cell responses (12, 13, 25). Both SARS-CoV-2–specific CD4^+^ and CD8^+^ T cells have been shown to be valuable components of protective immunity to SARS-CoV-2 and to correlate with less severe COVID-19 (11, 12, 26–28). SARS-CoV-2 antigen-specific CD4^+^ and CD8^+^ T cells can be detected as early as 3 days after symptom onset, and memory CD4^+^ and CD8^+^ T cells to SARS-CoV-2 remain detectable in most individuals for at least several months postinfection (12, 13, 18, 20, 21). Differences can be observed between SARS-CoV-2–specific CD4^+^ and CD8^+^ T cells, including cytokine production profiles and memory subsets (12, 13, 20, 21, 25, 26). This likely reflects the diverse array of effector and memory CD4^+^ and CD8^+^ T cells that are generated in response to SARS-CoV-2 infection, and the distinct roles played by these T cell subsets in shaping the antiviral response (11).

While certain T cell subsets like Th1 and cytotoxic CD4^+^ and CD8^+^ T cells may directly act on and/or eliminate virally infected cells, other T cell subsets like follicular helper CD4^+^ T cells (T_FH_) play a more indirect yet crucial role in refining B and T cell–mediated antiviral responses (27–30). Specifically, T_FH_ are important in nAb development, following infection (or vaccination) (11, 27, 28). For example, the changes observed in MBCs following SARS-CoV-2 infection point to a role for long-lived germinal center B cell–T cell interactions, in particular B cell–T_FH_ interactions, in the maturation of SARS-CoV-2–specific antibody responses (31).

Bamlanivimab is a neutralizing human IgG1 that can recognize the receptor binding domain (RBD) of the SARS-CoV-2 ancestral spike (S) protein and prevent RBD interaction with human angiotensin-converting enzyme 2, thereby blocking viral entry into host cells (32, 33). In November 2020, the administration of a single intravenous dose of 700 mg bamlanivimab was issued EUA by the FDA for the treatment of mild to moderate acute COVID-19 in the outpatient setting in adults at risk for severe COVID-19 and within 10 days from symptom onset (34). Here we present data on SARS-CoV-2–specific CD4^+^ and CD8^+^ memory T cell responses at study day 28 for 95 participants of the ACTIV-2/A5401 clinical trial with mild to moderate COVID-19 who received 700 mg of bamlanivimab (*n* = 46) or placebo (*n* = 49) on study day 0.

## Results

### Bamlanivimab treatment and SARS-CoV-2–specific CD4^+^ T cell frequencies.

This study examines early memory T cell responses in 95 persons who had acute COVID-19 approximately 1 month after these persons received either a single 700 mg intravenous dose of bamlanivimab (treatment; *n* = 46) or intravenous normal saline placebo control (placebo; *n* = 49). These persons received bamlanivimab or placebo on study day 0. Subsequently, peripheral blood was collected from these same persons on study day 28 to isolate and cryopreserve peripheral blood mononuclear cells (PBMCs) for memory T cell analyses. SARS-CoV-2–specific CD4^+^ and CD8^+^ memory T cell responses were measured for all participants at study day 28 and compared between treatment and placebo groups. PBMC samples with less than 85% cell viability (5/95 samples) upon thawing were excluded from analyses.

**Figure 1:**
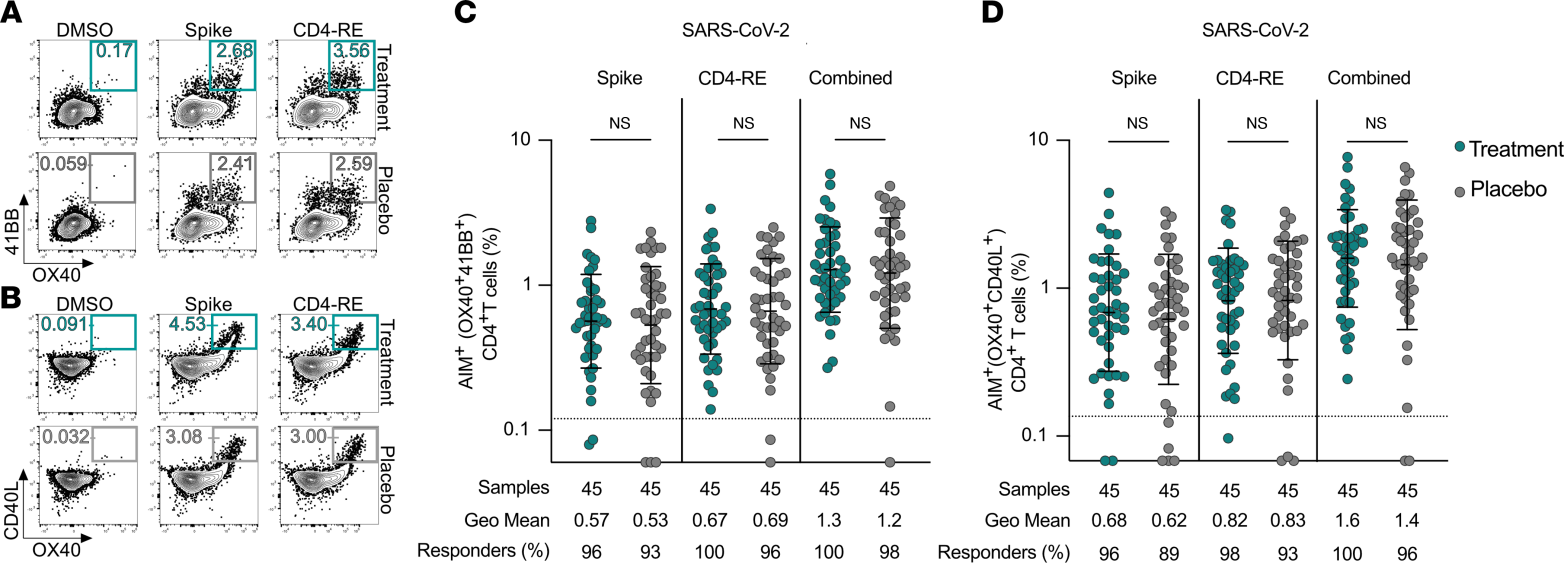
SARS-CoV-2–specific CD4^+^ T cell frequencies are equivalent following treatment with mAb or placebo. (**A**) Representative flow cytometry plots of SARS-CoV-2–specific CD4^+^ T cells (OX40^+^41BB^+^; see Supplemental Figure 1 for ancestral gating) at study day 28 in individuals who received mAb (upper, teal boxes) or placebo control (lower, gray boxes) for acute COVID-19. (**B**) Representative flow cytometry plots of SARS-CoV-2–specific CD4^+^ T cells (OX40^+^CD40L^+^; see Supplemental Figure 1 for ancestral gating) at study day 28 in individuals who received mAb (upper, teal boxes) or placebo control (lower, gray boxes) for acute COVID-19. (**C**) Percentage of background-subtracted spike, CD4-RE, or combined SARS-CoV-2–specific CD4^+^ T cells (surface OX40^+^41BB^+^, as percentage of CD4^+^ T cells) at study day 28 in individuals who received mAb (teal circles) or placebo control (gray circles) for acute COVID-19 by AIM assay following 24-hour stimulation of PBMCs with SARS-CoV-2 spike or CD4-RE megapool (MP). Combined AIM assay CD4^+^ T cell responses were calculated as the sum of the background-subtracted responses to individual (spike and CD4-RE) MPs. The dotted black line indicates the limit of quantification (LOQ). Baseline and nonresponders set at 0.5 of LOQ. Bars represent geometric mean with geometric standard deviation. Pairwise comparisons were made between equivalent stimulation conditions for treatment and placebo groups by Mann-Whitney nonparametric statistical testing. (**D**) Percentage of background-subtracted spike, CD4-RE, or combined SARS-CoV-2–specific CD4^+^ T cells (surface OX40^+^CD40L^+^, as percentage of CD4^+^ T cells) at study day 28 in individuals who received mAb (teal circles) or placebo control (gray circles) for acute COVID-19 by AIM assay following 24-hour stimulation with SARS-CoV-2 spike or CD4-RE MPs. Combined AIM assay CD4^+^ T cell responses were calculated as in **C**. The dotted black line, baseline, bars, and NS designation were calculated and defined as in **C**.

Frequencies of SARS-CoV-2-spike and non-spike (CD4-RE) specific CD4^+^ T cells were measured by activation-induced marker (AIM) assay (surface OX40^+^ and 41BB^+^ Figure 1, A and C, and Supplemental Figure 2A; supplemental material available online with this article; https://doi.org/10.1172/jci.insight.163471DS1; surface OX40^+^ and CD40L^+^ Figure 1, B and D, and Supplemental Figure 2B). Antigen-specific CD4^+^ T cell frequencies by surface expression of OX40 and either CD40L or 41BB positively correlated when plotted by these combinations of AIM markers, demonstrating that CD4^+^ T cell responses were comparable whether CD40L or 41BB was used in combination with OX40 for the identification of SARS-CoV-2–specific CD4^+^ T cells (Figure 1, C and D and Supplemental Figure 2C). At day 28, the majority (≥90%) of the participants from both the treatment and placebo groups had positive spike and non-spike SARS-CoV-2–specific CD4^+^ T cell responses (Figure 1, C and D and Supplemental Figure 1, A and B). There was no significant difference in the magnitude of antigen-specific CD4^+^ T cell responses between the treatment and placebo group based on percentages (Figure 1, C and D) or stimulation index (SI; Supplemental Figure 2, A and B) by Mann-Whitney test. The percentage of individuals producing detectable responses to spike, non-spike, or all epitopes was 89% to 100% and not significantly different between the treatment and placebo groups for any stimulation condition (Fisher’s exact test *P* value 0.43 to >0.99 for spike, CD4-RE and combined antigen-specific CD4^+^ responses by AIM; Figure 1, C and D). All the treatment group (45/45) and nearly all the placebo group (44/45) participants (88–89/90 total) had measurable SARS-CoV-2–specific CD4^+^ T cell responses by at least 1 AIM measure (“combined,” Figure 1, C and D).

### SARS-CoV-2–specific CD4^+^ T cell functional responses.

**Figure 2:**
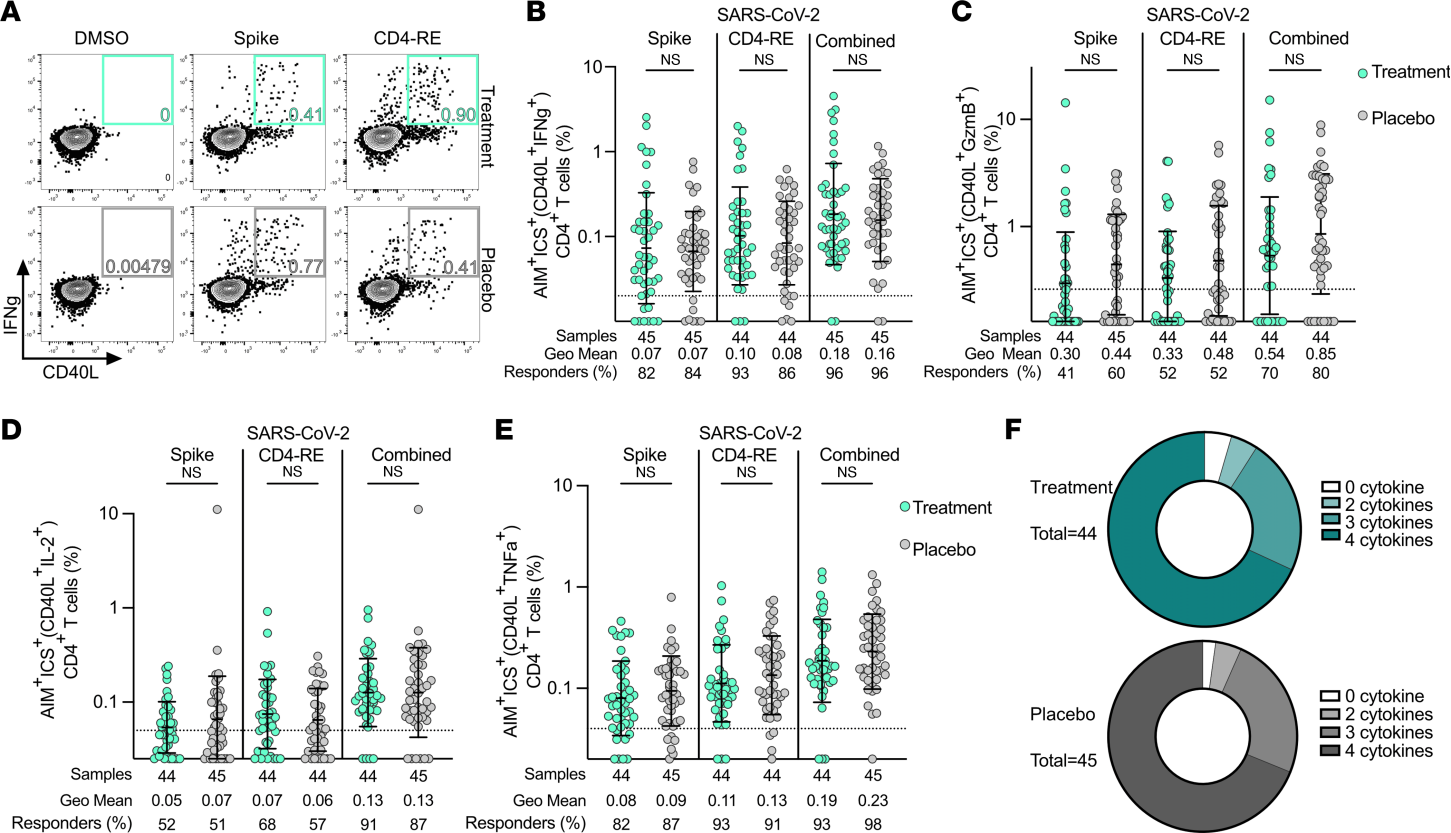
SARS-CoV-2–specific CD4^+^ T cell functionality is equivalent following treatment with mAb or placebo. (**A**) Representative flow cytometry plots of SARS-CoV-2–specific, IFN-γ^+^CD4^+^ T cells (CD40L^+^IFN-γ^+^; see Supplemental Figure 1 for ancestral gating) at study day 28 in individuals who received mAb (upper, light green boxes) or placebo control (lower, gray boxes) for acute COVID-19. (**B**–**E**) Percentage of background-subtracted spike, CD4-RE, or combined SARS-CoV-2–specific, cytokine-producing CD4^+^ T cells (surface CD40L^+^ intracellular cytokine positive, as percentage of CD4^+^ T cells) at study day 28 in the treatment (light green circles) and placebo (gray circles) groups by hybrid AIM+ICS following 24-hour stimulation with SARS-CoV-2 spike or CD4-RE MPs for IFN-γ (**B**), GzmB (**C**), IL-2 (**D**), and TNF-α (**E**). Combined AIM+ICS assay cytokine-producing, antigen-specific CD4^+^ T cell responses were calculated for each condition as the sum of the background-subtracted responses to individual (spike and CD4-RE) MPs. The dotted black line indicates the LOQ. Baseline set at 0.5 of LOQ. Bars represent geometric mean with geometric standard deviation. Pairwise comparisons were made between equivalent stimulation conditions for treatment and placebo groups by Mann-Whitney nonparametric statistical testing. (**F**) Donut charts representing the proportion of antigen-specific CD4^+^ T cells producing 0–4 cytokines at day 28.

Functionality of SARS-CoV-2–specific CD4^+^ T cells at day 28 was assessed by a combination of surface AIM marker expression with intracellular cytokine staining (AIM+ICS) following stimulation with spike and non-spike peptide MPs (CD4^+^ surface CD40L^+^ plus IFN-γ, TNF-α, Granzyme B [GzmB], and/or IL-2; Figure 2, A–F, and Supplemental Figure 3). There was no difference in polyfunctionality of SARS-CoV-2–specific antiviral CD4^+^ T cells observed between the treatment and placebo groups based on cytokine production (Figure 2F). Overall, 96% of treatment and placebo group participants had SARS-CoV-2–specific IFN-γ^+^CD4^+^ T cell responses; 82% of treatment and 84% of placebo participants had IFN-γ^+^CD4^+^ T cell responses detectable to spike epitopes, and 93% of treatment and 86% of placebo participants had IFN-γ^+^CD4^+^ T cell responses detectable to non-spike epitopes (Figure 2B). IFN-γ^+^CD4^+^ T cell response magnitudes were not significantly different between the treatment and placebo groups for any stimulation condition, whether comparing percentages (Figure 2B) or SI (Supplemental Figure 3A), by Mann-Whitney test. SARS-CoV-2–specific CD4^+^ T cells most often demonstrated production of IFN-γ and TNF-α (Figure 2, B and E). GzmB (Figure 2C) and IL-2 (Figure 2D) production was also seen. Cytokine production by antigen-specific CD4^+^ T cells predominantly exhibited a Th1 profile, as would be anticipated for acute COVID-19. SARS-CoV-2–specific CD4^+^ T cells from trial participants who received mAb therapy did not demonstrate less cytokine production than those who received placebo for any of the cytokines measured (Figure 2 and Supplemental Figure 3).

### SARS-CoV-2–specific circulating T_FH_ cells.

**Figure 3:**
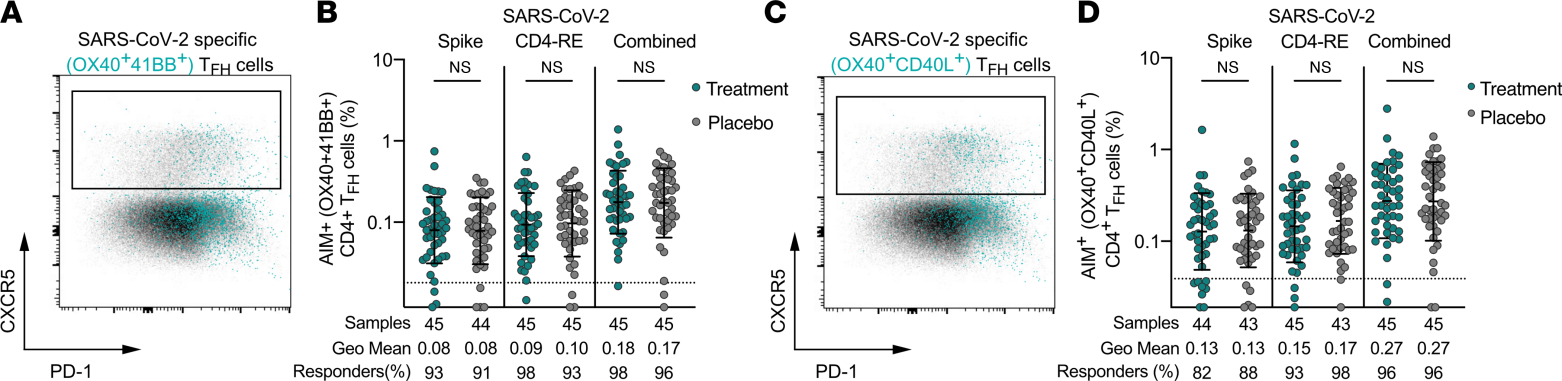
SARS-CoV-2–specific circulating T_FH_ are equivalent following treatment with mAb or placebo. (**A** and **C**) Representative flow plots for antigen-specific T_FH_ (CXCR5 × PD-1 gated on all CXCR5^+^AIM^+^CD4^+^); AIM markers included OX40 and 41BB (**A**) or OX40 and CD40L (**C**). PD-1, programmed cell death 1. (**B** and **D**) Percentage of spike, CD4-RE, or combined SARS-CoV-2–specific circulating T_FH_ (CXCR5^+^ as percentage of AIM^+^CD4^+^ T cells) at study day 28 in individuals who received mAb (teal circles) or placebo control (gray circles) for acute COVID-19 by AIM assay following 24-hour stimulation of PBMCs with SARS-CoV-2 spike or CD4-RE MPs; AIM markers included OX40 and 41BB (**B**) or OX40 and CD40L (**D**). Combined T_FH_ responses were calculated as the sum of the T_FH_ specific to the individual (spike and CD4-RE) MPs. Bars represent geometric mean with geometric standard deviation. Pairwise testing by Mann-Whitney. Dotted line represents LOQ. Baseline set to 0.5 of LOQ.

SARS-CoV-2–specific CD4^+^ circulating T_FH_ (defined by CXCR5 positivity) at day 28 were assessed by AIM following 24-hour stimulation with viral spike and non-spike peptides (Figure 3). Here circulating T_FH_ are defined as CXCR5^+^ antigen-specific CD4^+^ T cells in order to capture all activated and resting memory circulating T_FH_ responses to SARS-CoV-2, rather than solely responses of activated circulating T_FH_, which would additionally be defined by positive (PD-1^+^) to high (PD-1^hi^) PD-1 surface expression. Both the treatment and placebo groups had similar T_FH_ SARS-CoV-2–specific CD4^+^ T cell frequencies to spike and non-spike epitopes at day 28 (Figure 3, B and D, and Supplemental Figure 4). No significant differences in antigen-specific T_FH_ frequencies or positivity were observed between the treatment and placebo groups by Mann-Whitney test (Figure 3, B and D). Similar results were obtained when antigen-specific T_FH_ were measured by SI (Supplemental Figure 4, A and B). Between 82% and 98% of individuals had antigen-specific T_FH_, with no significant difference in positive response rates by Fisher’s exact tests (*P* values 0.56 to >0.99 for antigen-specific spike, non-spike, and combined T_FH_ responses; Figure 3, B and D). Surface expression of CCR6 and CXCR3 on AIM^+^ antigen-specific T_FH_ cells was also examined (Supplemental Figure 4, C and D) as the presence or absence of these markers may be indicators of T_FH_ functionality important for adaptive immunity to SARS-CoV-2. T_FH_ with CCR6 and CXCR3 surface expression have been demonstrated to play a role in lung homing and germinal center and rapid anamnestic (recall) responses, respectively (13, 27, 35). Memory T_FH_ negative for surface CXCR3 expression are associated with high-quality antibody and germinal center responses (36, 37). No significant difference in the surface expression patterns of the chemokine receptors CCR6 and CXCR3 was observed on antigen-specific T_FH_ cells following mAb therapy versus placebo by Mann-Whitney test (Supplemental Figure 4, C and D).

### SARS-CoV-2–specific CD4^+^ T cell memory subsets.

**Figure 4:**
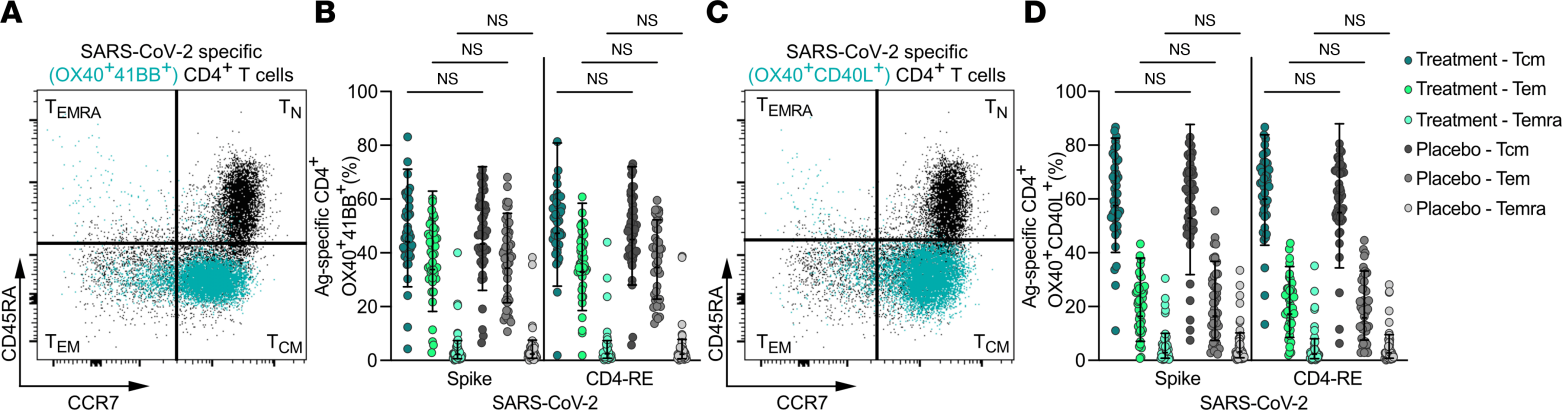
SARS-CoV-2–specific CD4^+^ memory T cell subsets are equivalent following receipt of mAb or placebo. (**A** and **C**) Representative flow cytometry plots of total circulating naive and memory CD4^+^ T cell subsets (black dots) and proportion of naive/memory cells that are SARS-CoV-2–specific CD4^+^ T cells by AIM (teal overlay; OX40^+^41BB^+^ in **A**, OX40^+^CD40L^+^ in **C**; see Supplemental Figure 1 for ancestral gating). (**B** and **D**) Percentage of SARS-CoV-2–specific CD4^+^ T cells that are T_cm_, T_em_, and T_emra_ at study day 28 in individuals who received mAb (3 shades of teal/green circles) or placebo control (3 shades of gray circles) for acute COVID-19 by AIM (**B**, OX40^+^41BB and **D**, OX40^+^CD40L^+^) following 24-hour stimulation with SARS-CoV-2 spike or CD4-RE MPs. T cell subtype (T_cm_, T_em_, and T_emra_) was assigned based on surface expression of CCR7 and/or CD45RA, as in **A** and **C**. Bars represent geometric mean with geometric standard deviation. Equivalent memory T cell populations for treatment and placebo groups were compared by Kruskal-Wallis tests with Dunn’s post hoc correction for multiple comparisons.

SARS-CoV-2–specific CD4^+^ T cells in the treatment and placebo groups were classified into memory subsets based on CD45RA and CCR7 surface expression patterns following stimulation with SARS-CoV-2 spike and non-spike epitope-containing MPs by AIM (Figure 4, A–D). Similar to prior studies of adaptive immunity during the convalescent phase of COVID-19 (13, 21), T_cm_ and T_em_ were the most frequent CD4^+^ T cell memory phenotypes observed (Figure 4, B and D). Similar findings regarding memory CD4^+^ T cell subset frequencies and subsets were observed by AIM+ICS (Supplemental Figure 5, A and B). SARS-CoV-2–specific CD4^+^ T cell memory phenotype frequencies at day 28 posttreatment were not altered by bamlanivimab (Figure 4, B and D, and Supplemental Figure 5, A and B).

### SARS-CoV-2–specific CD8^+^ T cell frequencies and functional responses.

**Figure 5:**
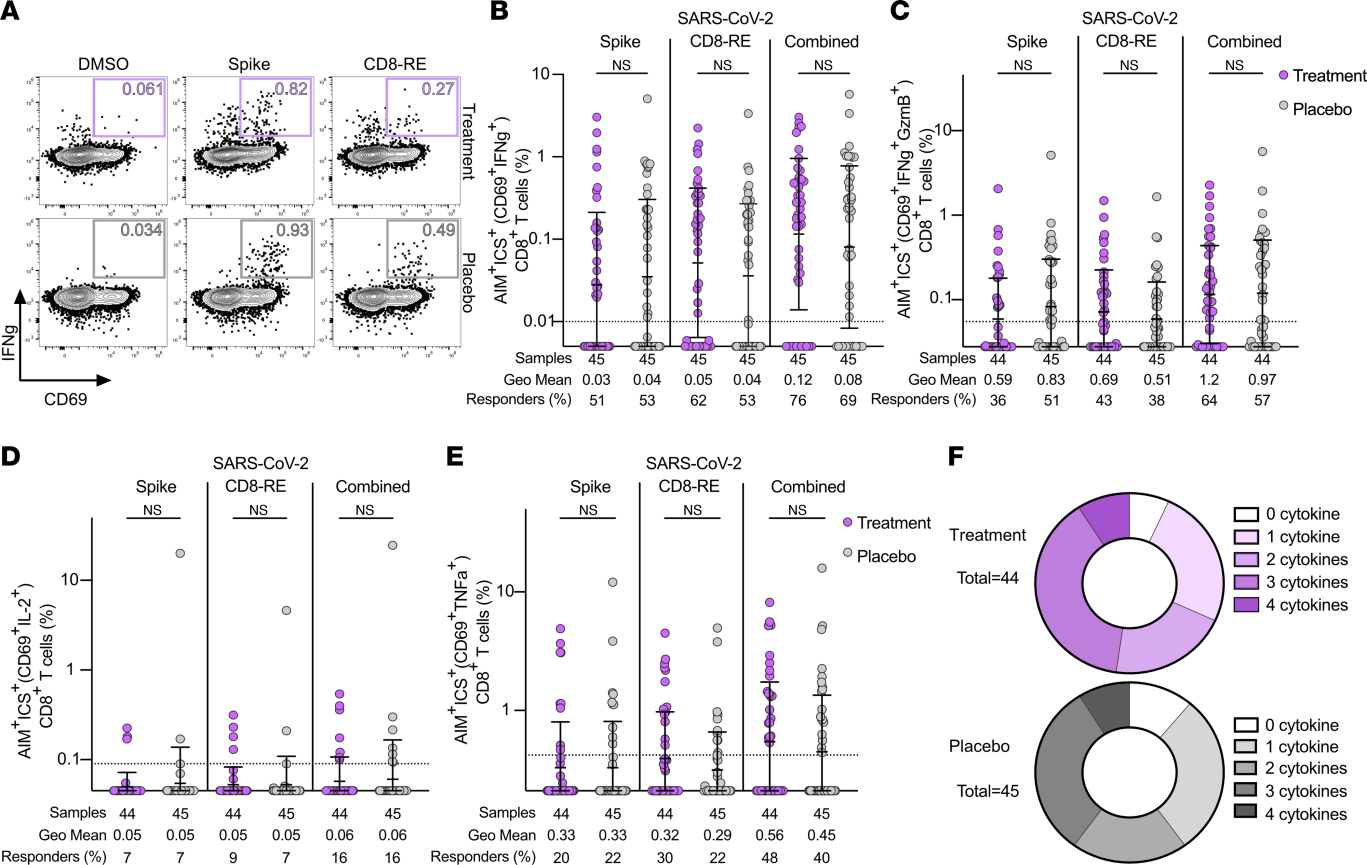
SARS-CoV-2–specific CD8^+^ T cell responses and functionality are equivalent following treatment with mAb or placebo. (**A**) Representative flow cytometry plots of SARS-CoV-2–specific, IFN-γ^+^CD8^+^ T cells (CD40L^+^IFN-γ^+^; see Supplemental Figure 1 for ancestral gating) at study day 28 in individuals who received mAb (upper, lavender boxes) or placebo control (lower, gray boxes) for acute COVID-19. (**B**–**D**) Percentage of background-subtracted spike, CD8-RE, or combined SARS-CoV-2–specific, cytokine-producing CD8^+^ T cells (surface CD69^+^ intracellular cytokine positive, as percentage of CD8^+^ T cells) at study day 28 in individuals who received mAb (lavender circles) or placebo control (gray circles) for acute COVID-19 by hybrid AIM+ICS following 24-hour stimulation with SARS-CoV-2 spike or CD4-RE MPs for IFN-γ (**B**), IFN-γ + GzmB (**C**), IL-2 (**D**) and TNF-α (**E**). Combined AIM+ICS assay cytokine-producing, antigen-specific CD8^+^ T cell responses were calculated for each condition as the sum of the background-subtracted responses to individual (spike and CD4-RE) MPs. The dotted black line indicates the LOQ. Baseline set at 0.5 of LOQ. Bars represent geometric mean with geometric standard deviation. Mann-Whitney test. (**F**) Donut charts representing the proportion of antigen-specific CD8^+^ T cells producing 0–4 cytokines at day 28 in the treatment and placebo groups.

SARS-CoV-2–specific CD8^+^ T cell responses at day 28 were also evaluated in the treatment and placebo groups by AIM (surface CD69^+^ and 41BB^+^; Supplemental Figure 6, C–E) and AIM+ICS (surface CD69^+^ and intracellular IFN-γ^+^; Figure 5, A and B, and Supplemental Figure 6A). The use of AIM+ICS allowed for simultaneous assessment of CD8^+^ T cell specificity and functionality and proved more sensitive than AIM alone for the detection of SARS-CoV-2 spike- and non-spike–specific CD8^+^ T cells (Figure 5B and Supplemental Figure 6). Over half of participants (51%–53%) made detectable CD8^+^ T cell responses to spike and 53% to 62% made detectable CD8^+^ T cell responses to non-spike epitopes (Figure 5B). Overall, 51% to 62% of participants generated antiviral CD8^+^ T cell responses to spike or non-spike epitopes (Figure 5B), and 69% to 76% made detectable responses to spike and non-spike epitopes combined. There was no significant difference between CD8^+^ T cell response rates in treatment and placebo groups (69% compared to 76%, Fisher’s exact test *P* = 0.64; Figure 5B). There was also no significant difference in the magnitude of antigen-specific CD8^+^ T cell responses between the treatment and placebo group based on percentages (Figure 5B) or SI (Supplemental Figure 6A).

In addition to IFN-γ production, antigen-specific CD8^+^ T cells produced additional cytokines, including GzmB (in conjunction with IFN-γ) (Figure 5C), IL-2 (Figure 5D), and TNF-α (Figure 5E), in response to stimulation with SARS-CoV-2 peptides. As expected, IL-2 production by antigen-specific CD8^+^ T cells was rare (Figure 5D). There was no difference in cytokine production by antiviral CD8^+^ T cells observed between the treatment and placebo groups based on the production of single or multiple cytokines by SARS-CoV-2–specific CD8^+^ T cells (Fisher’s exact test *P* values 0.19 to 0.98 for all cytokines and stimulation conditions; Figure 5, B–F).

### SARS-CoV-2–specific CD8^+^ T cell memory subsets.

**Figure 6:**
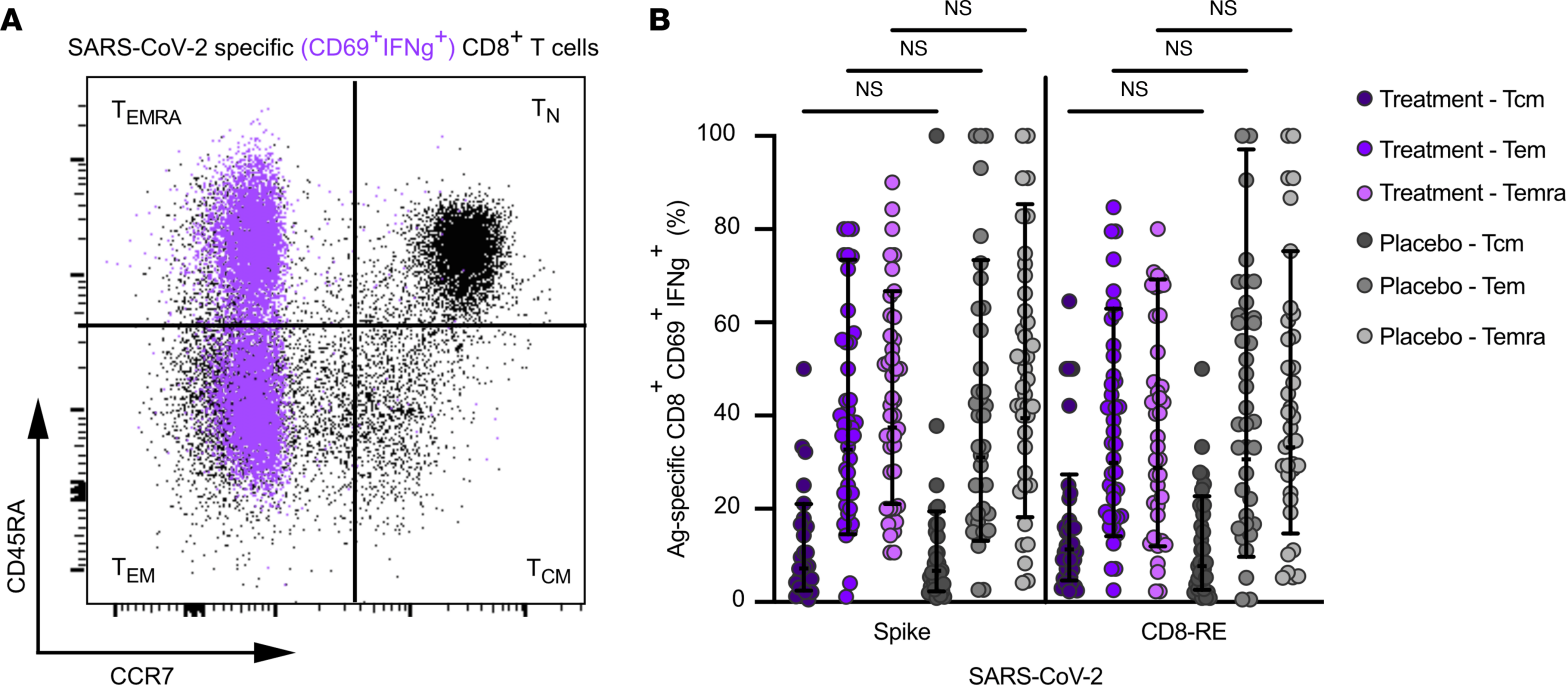
SARS-CoV-2–specific CD8^+^ memory T cell subsets are equivalent following receipt of mAb or placebo. (**A**) Representative flow cytometry plots of total circulating naive and memory CD8^+^ T cell subsets (black) and proportion of naive/memory cells that are SARS-CoV-2–specific CD8^+^ T cells by AIM+ICS (purple overlay, CD69^+^IFN-γ^+^; see Supplemental Figure 1 for ancestral gating). (**B**) Percentage of SARS-CoV-2–specific (surface CD69^+^ intracellular IFN-γ^+^) CD8^+^ T cells that are T_cm_, T_em_, and T_emra_ at study day 28 in individuals who received mAb (3 shades of purple circles) or placebo control (3 shades of gray circles) for acute COVID-19 by hybrid AIM+ICS following 24-hour stimulation with SARS-CoV-2 spike or CD8-RE MPs. T cell subtype (T_cm_, T_em_, T_emra_) was assigned based on surface expression of CCR7 and/or CD45RA, as in **A**. Bars represent geometric mean with geometric standard deviation. Equivalent memory T cell populations for treatment and placebo groups were compared by Kruskal-Wallis tests with Dunn’s post hoc correction for multiple comparisons.

SARS-CoV-2–specific CD8^+^ T cell memory subsets were evaluated at day 28 (Figure 6, A and B). No significant differences were observed in the memory CD8^+^ T cell populations between the treatment and placebo groups (Figure 6B). Following mAb or placebo therapy, antigen-specific memory CD8^+^ T cells generated in response to acute COVID-19 were predominantly T_em_ and T_emra_ (Figure 6B), consistent with previous reports of untreated persons with COVID-19 (13, 21).

### Baseline and day 28 SARS-CoV-2–specific IgG antibody responses.

**Figure 7:**
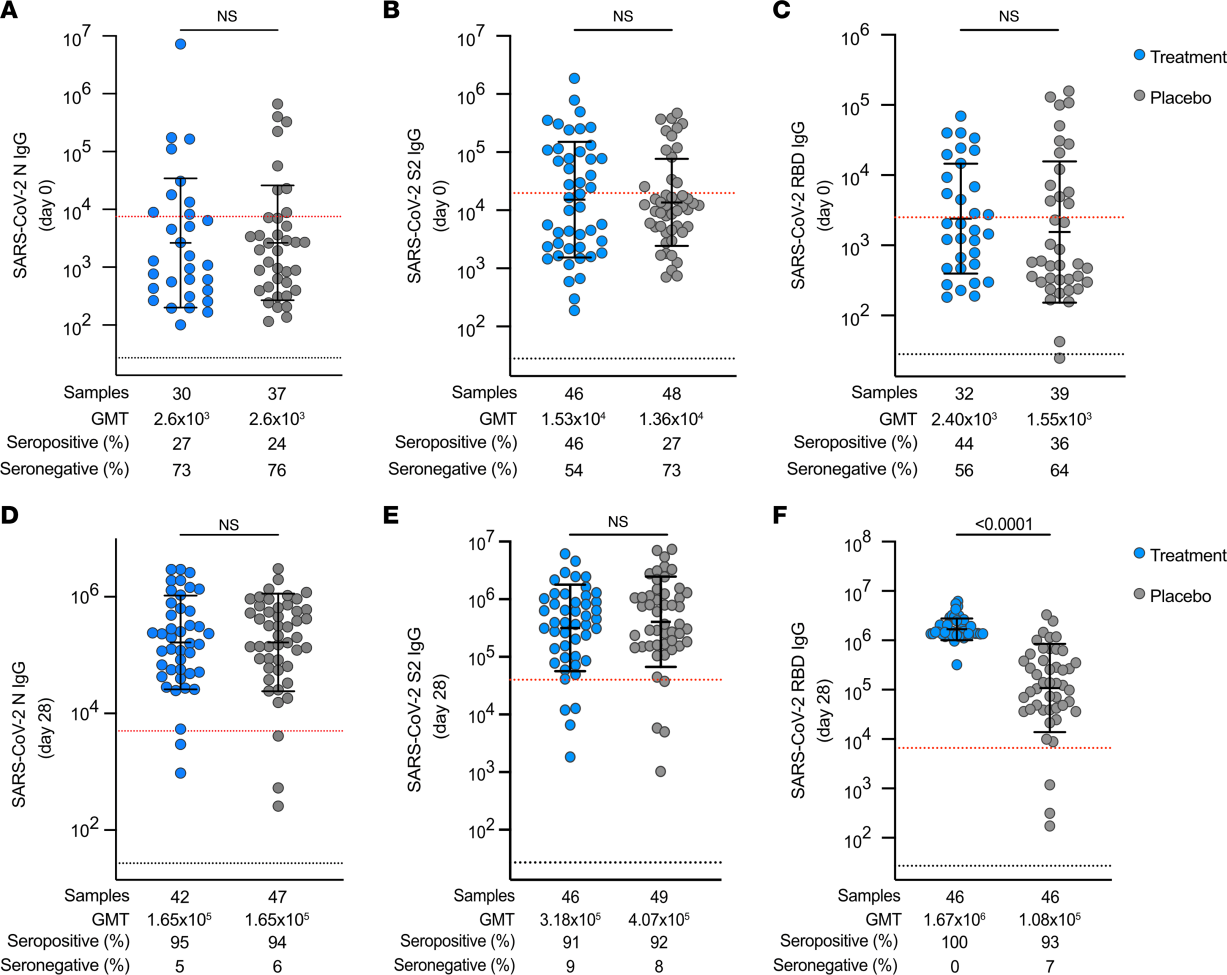
SARS-CoV-2–specific baseline IgG titers and seropositivity are similar among treatment and placebo groups, and non-RBD IgG titers and seropositivity rates at day 28 are similar among treatment and placebo group participants. (**A**–**C**) Baseline IgG titers for ancestral SARS-CoV-2 N (in **A**), S2 (in **B**), and RBD (in **C**) at study entry (day 0) in individuals who received bamlanivimab (blue circles) versus placebo (gray circles) in AU/mL. (**D**–**F**) Day 28 IgG titers for ancestral SARS-CoV-2 N (in **D**), S2 (in **E**), and RBD (in **F**) in treatment (blue circles) and placebo (gray circles) groups. Dotted black line indicates lower LOQ of detection (titer) for Bio-Plex (N, S2, RBD) serologic assays. Dotted red line indicates cutoff for positivity for Bio-Plex assays; titer results were generated using a standard curve generated by standards provided by the manufacturer (out-of-range titers were excluded from analyses). MFI-based positivity was set based on results generated using prepandemic, SARS-CoV-2–uninfected control samples. See also Supplemental Figure 7. GMT, geometric mean titer.

Blood was drawn prior to administration of bamlanivimab or placebo for antibody response assessments at study enrollment (baseline, day 0) including IgG against the SARS-CoV-2 nucleocapsid (N), ancestral spike S2 domain (S2), and RBD. Antibody titers were measured using the Bio-Plex Pro Human SARS-CoV-2 serology IgG assay (Bio-Rad Laboratories, Inc., Life Sciences Group). There were no significant differences in baseline serostatus between the treatment or placebo group participants for whom T cell responses were evaluated (as would be expected because assignment was randomized). At day 0, SARS-CoV-2 IgG titers against N, ancestral S2, and RBD were not significantly different between the treatment and placebo groups (Figure 7, A–C). Most participants were seronegative and approximately equal numbers of participants from both groups were seropositive for RBD IgG at baseline (Figure 7C). Antibody responses to N, S2, and RBD were again measured at day 28 by the Bio-Plex Pro Human IgG assay (Figure 7, D–F). Similar percentages of the treatment and placebo groups were seropositive for N and S2 (Figure 7, D and E) at day 28. Although S2 IgG titers were not significantly different between the groups at day 28 (Figure 7E), RBD IgG titers were significantly higher in the treatment group at day 28 (Figure 7F), likely due to the long half-life and high levels of circulating bamlanivimab remaining in the blood of treatment group participants at day 28 (37). At day 28, nAb titers in the treatment group would be anticipated to reflect a mixture of endogenous anti-SARS-CoV-2 nAb as well as bamlanivimab, making comparisons of endogenous nAb titers between the treatment and placebo groups at day 28 challenging to interpret. Thus, day 28 nAb titer data were not included in this study.

## Discussion

Given that both SARS-CoV-2 infection and use of mAb therapy for the treatment of acute COVID-19 are prevalent, understanding the long-term effects of mAb therapy on adaptive immunity to SARS-CoV-2 is of high importance and may influence clinical decision-making and public health policy. For example, if mAb therapy were found to negatively impact humoral immunity to SARS-CoV-2 developed during acute COVID-19, resulting in less durable or lower nAb titers, then it would be important to understand how this effect could impact individual risk for reinfection and responses to COVID-19 vaccination to know if recommendations for vaccination should differ for individuals treated with mAb therapy. In such a scenario, it would also be informative to determine if the cellular immune response could compensate for diminished humoral immunity. Given our prior findings that suggest that cellular immunity to SARS-CoV-2 can mitigate severe COVID-19 (12), we sought to examine the impact of mAb therapy for acute COVID-19 on cellular memory immunity to SARS-CoV-2.

We analyzed SARS-CoV-2–specific CD4^+^ and CD8^+^ T cell responses from the peripheral blood of 95 persons who had acute COVID-19 following infection with ancestral SARS-CoV-2 and who participated in a randomized, placebo-controlled trial (6). In the trial, participants were treated with mAb with activity against ancestral SARS-CoV-2 (34), bamlanivimab, or placebo (6). Antigen-specific circulating T_FH_ and CD4^+^ and CD8^+^ memory T cell subsets were detected in most participants at 28 days after bamlanivimab or placebo. Study day 28 was the latest prevaccination time point available for analysis of circulating SARS-CoV-2 memory T cell responses for this cohort. No significant differences were observed in SARS-CoV-2–specific CD4^+^ or CD8^+^ T cell magnitude, functionalities, or breadth in trial participants at 28 days after receipt of mAb versus placebo.

It has been hypothesized that SARS-CoV-2–specific mAb administration may result in diminished antiviral T cell responses due to enhanced viral clearance and reduced viral burden following viral neutralization by the mAb, resulting in decreased viral protein antigen present to prime antiviral T cell responses. Alternatively, it has been posited that mAb therapy could enhance SARS-CoV-2–specific adaptive immunity via a “vaccinal effect,” as has been proposed for other antibody-based therapeutics. Our data do not support either of these hypotheses (4, 7–10). Antigen-specific T cell responses were found to be similarly directed against both spike and non-spike epitopes and to be of both similar quality and quantity about 1 month following bamlanivimab or placebo therapy for acute COVID-19. These findings are in accordance with prior studies that have found that most individuals in the convalescent phase of mild to moderate COVID-19 have SARS-CoV-2–specific CD4^+^ and CD8^+^ T cells that recognize multiple viral antigens across the SARS-CoV-2 ORFeome (13, 20, 25). Antigen-specific CD4^+^ memory T cells formed in response to SARS-CoV-2 infection are primarily T_cm_ and T_em_, whereas antigen-specific CD8^+^ memory T cells are primarily T_em_ and T_emra_, and here we detected no impact of mAb treatment on SARS-CoV-2–specific T cell memory phenotypes.

SARS-CoV-2–specific T_FH_ cells had similar frequencies of CCR6 and/or CXCR3 surface expression at day 28 regardless of mAb therapy administration. As mentioned above, surface expression patterns of the chemokine receptors CCR6 and CXCR3 on AIM^+^ antigen-specific T_FH_ cells may be of particular interest when evaluating adaptive immunity to a respiratory pathogen like SARS-CoV-2. Given that surface expression of CCR6 and CXCR3 on SARS-CoV-2–specific T_FH_ did not differ significantly, it is anticipated that individuals who received bamlanivimab would not have inferior T_FH_-influenced SARS-CoV-2–specific adaptive immunity.

The similarity in the T cell responses would be consistent with the mAb-treated and placebo-treated groups experiencing substantially similar total exposure to viral protein antigens, resulting in sufficient antigen presentation and priming of T cells. Studies of other antiviral mAb therapeutics have demonstrated that humoral immunity may be diminished by mAb administration while cellular immunity is not (7). Data from clinical trials of the monoclonal casirivimab and imdevimab antibody cocktail (Regeneron Pharmaceuticals) demonstrated that reductions in SARS-CoV-2 viral levels were transient and modest (4). Enhanced viral clearance following this mAb therapy was most pronounced in individuals who were seronegative and/or had high viral loads at the time of mAb administration, resulting in a median viral load reduction of approximately 3-fold after 7 days (4, 11). Individuals who already had evidence of adaptive immunity to SARS-CoV-2, measured by SARS-CoV-2 antibody seropositivity, and received REGN-CoV mAb therapy displayed minimal differences in viral clearance (4). The participants from the ACTIV-2/A5401 bamlanivimab 700 mg and placebo control groups studied here were comparable in terms of their baseline seropositivity. The majority of participants in both groups were seronegative, suggesting that the study participants had not developed adaptive immune responses to SARS-CoV-2 prior to administration of mAb or placebo and that the adaptive immunity findings reported here were not confounded by differences in baseline humoral immunity between the study groups. Similar to REGN-CoV mAb, bamlanivimab was also shown to have transient and modest antiviral activity in the setting of mild to moderate outpatient acute COVID-19 (4, 6). The primary outcomes analysis for the ACTIV-2/A5401 clinical trial found that administration of a single intravenous dose of 700 mg of bamlanivimab at study entry resulted in lower nasopharyngeal (NP) SARS-CoV-2 viral RNA at study day 3 in the treatment group compared with the placebo group (37). NP SARS-CoV-2 viral RNA levels in the treatment group also demonstrated more rapid reduction in viral RNA levels by decay modeling versus the placebo group (6).

Although this study focused on understanding the impact of bamlanivimab therapy on SARS-CoV-2–specific memory CD4^+^ and CD8^+^ T cell responses, we were also able to examine SARS-CoV-2–specific IgG titers in the same participants. There were no differences in non-RBD IgG titers between the mAb and placebo groups at day 28. In contrast, it was found that following a higher (4,200 mg) prophylactic dose of bamlanivimab, individuals without a history of SARS-CoV-2 infection had lower antibody titers after 2 doses of a COVID-19 mRNA vaccine than individuals who had received placebo control (5). However, antibody titers differed between the groups by 2-fold or less, which was considered a clinically insignificant difference (5). It is possible that at higher doses bamlanivimab has a greater impact on humoral immunity to SARS-CoV-2 but without significantly impairing responses to subsequent vaccination.

Our study had a number of limitations, including cross-sectional design, small study population size, and potential lack of generalizability. This study does not directly assess the protective nature or durability of the SARS-CoV-2–specific T cell responses that were generated by the individuals who were studied. This study examines adaptive immunity in individuals with mild to moderate, outpatient COVID-19 with normal leukocyte counts but with other risk factors for progression to severe COVID-19. Our findings may not be reflective of all individuals with acute COVID-19; in particular, individuals may have specific forms of immunocompromise or immunosuppression, or other risk factors for severe COVID-19, that are not represented by our study population or could not be assessed by this study.

This study is not an efficacy study. The efficacy of bamlanivimab against COVID-19 caused by ancestral SARS-CoV-2 was assumed based on published data, including the primary outcomes from ACTIV-2/A5401 and other clinical trials (6, 34, 38–40). Bamlanivimab represents just one of the many mAb (single agent and combination) therapeutics developed for the treatment of acute COVID-19 that was given EUA by the US FDA (7). Bamlanivimab has not had EUA as a single-agent therapeutic for COVID-19 since April 2021. Bamlanivimab is available as a combination therapeutic together with etesevimab. Bamlanivimab/etesevimab is not effective against the Omicron variant, and Eli Lilly and Company has voluntarily agreed to have the EUA removed for the use of bamlanivimab/etesevimab in regions where SARS-CoV-2 infection is likely to be caused by Omicron or other nonsusceptible (sub-)variants (41). Our findings may not be applicable to COVID-19 caused by SARS-CoV-2 variants not assessed in this study. Additionally, it is possible that other SARS-CoV-2–specific mAb therapeutics may impact adaptive immunity to SARS-CoV-2 differently than a single 700 mg intravenous dose of bamlanivimab. Our findings may not be generalizable to other bamlanivimab doses or other therapeutics for COVID-19. It will be important for future studies to examine the impact of other anti-SARS-CoV-2 mAb therapies, including the impact of both individual mAbs and combinations of mAbs present in mAb cocktails, on adaptive immunity to SARS-CoV-2. When evaluating humoral immunity in the setting of mAb administration, the half-life of the mAb and the potential for circulating mAb to confound both binding and neutralizing antibody titers for regions of the spike protein that are targeted by the mAb must be considered.

Overall, our data demonstrate that most immunocompetent individuals with mild to moderate acute COVID-19 indeed develop robust antiviral memory T cell responses, regardless of administration of a SARS-CoV-2–targeted mAb therapy. Specifically, our findings are reassuring that individuals who received bamlanivimab during acute COVID-19 caused by ancestral SARS-CoV-2 were able to form antigen-specific, polyfunctional, antiviral Th1, T_FH_, and memory CD4^+^ T cells as well as antigen-specific polyfunctional effector and memory CD8^+^ T cells at similar levels to individuals who received only placebo. Considering the multiplicity of roles that T cells play in protective immunity to SARS-CoV-2, our findings may help influence clinical decision-making regarding the use of mAb-based therapeutics for acute COVID-19.

## Methods

### Study population and trial.

Participants in this study represent a subset of participants from the ACTIV-2/A5401 phase II/III clinical trial. All the bamlanivimab 700 mg group and comparison placebo control group participants from the corresponding complete ACTIV-2/A5401 study groups with available clinical data (e.g., treatment assignment, demographics, risk for severe COVID-19; see Table 1) and available PBMC samples collected for immunologic outcomes testing were included in this study. PBMC samples from approximately 41% of participants from the bamlanivimab 700 mg and comparison placebo groups were available for this study. Participants were enrolled in the United States between October and November 2020, a period in which COVID-19 in the United States could be attributed to ancestral SARS-CoV-2. The groups were comparable in terms of risk for progression to severe COVID-19, baseline serostatus, and time from COVID-19 symptom onset to enrollment. Please see Table 1 for additional details about the participants. ACTIV-2/A5401 is an ongoing, multicenter phase II/III randomized controlled trial designed to evaluate the safety and efficacy of therapeutics for acute COVID-19 in nonhospitalized adults. Inclusion criteria included adults age 18 years or older with documented SARS-CoV-2 infection by FDA-authorized antigen or molecular testing within 7 days prior to study entry and no more than 10 days of symptoms at the time of enrollment. Participants were assigned to bamlanivimab (treatment) or placebo groups at a 1:1 ratio. Randomization was stratified by both time from symptom onset (less than or equal to 5 days after symptom onset versus greater than 5 days) and risk of progression to severe COVID-19 (low versus high, based on age and comorbid medical conditions). Specifically, individuals assigned to the “high-risk” group for progression to severe COVID-19 were age ≥ 55 years and/or had 1 or more of the following medical conditions: chronic lung disease or moderate to severe asthma, body mass index > 35 kg/m^2^, hypertension, cardiovascular disease, diabetes, chronic kidney disease, or chronic liver disease (6). Additional information is available at ClinicalTrials.gov (Identifier: NCT04518410). The complete protocol, including complete eligibility criteria for the study and additional clinical data, can be found in the supplementary materials for the primary outcomes manuscript (6).

### PBMCs and viability-based quality control.

Peripheral blood was collected from ACTIV-2/A5401 participants at assigned study days. Serum and PBMCs were isolated from whole blood using standard operating procedures. PBMCs were cryopreserved and stored in liquid nitrogen prior to use. Just prior to use, cryopreserved PBMCs were thawed at 37°C and then resuspended in prewarmed complete RPMI medium with 5% human AB serum (Gemini Bioproducts) and benzonase. Following washing, cell counts and viability were assessed on a Muse Cell Analyzer (Luminex) using the Muse Count & Viability Kit. Additional medium was added to the PBMCs to achieve a final concentration of 100,000 PBMCs/100 μL. PBMC samples with less than 85% viability were excluded from analyses. Additional quality control metrics based on T cell responses to the AIM and AIM+ICS assays were also applied, as described below.

### AIM assay.

PBMCs were cultured for 24 hours at 37°C in an incubator with 5% CO_2_ in the presence of DMSO (negative control; equimolar amount as DMSO vehicle for MPs), Staphylococcal enterotoxin B (SEB; positive control, 1 μg/mL), or SARS-CoV-2 MPs (25, 42, 43) containing spike (25, 43) or non-spike epitopes (CD4-RE dominant and subdominant MPs) (42) (1 μg/mL per MP). PBMCs were plated on a 96-well plate with 1 × 10^6^ PBMCs per MP stimulation well and between 0.5 × 10^6^ and 1 × 10^6^ PBMCs per control well; DMSO controls were plated in duplicate except in rare cases where PBMCs were limited. Prior to stimulation, 0.5 μg/mL anti–human CD40 mAb (Miltenyi Biotec) blocking antibody was added to the PBMCs, and the plate was incubated at 37°C for 15 minutes in an incubator with 5% CO_2_. Chemokine receptor antibodies were also added to the wells on day 1 (see Supplemental Table 1 for antibodies used in the AIM assay). Following the 24-hour incubation, the PBMCs were centrifuged, washed with PBS, stained with LIVE/DEAD Fixable Blue (Invitrogen), diluted 1:1,000 in PBS with Fc block (5 μL/sample; BD Biosciences [BD]) for 15 minutes at room temperature, washed with FACS buffer (3% FBS in Dulbecco’s PBS without calcium or magnesium), surface stained (see Supplemental Table 1 for surface staining panel) for 30 minutes at 4°C, washed with FACS buffer, fixed with Cytofix Fixation Buffer (BD) at 4°C for 20 minutes, washed twice with Stain Buffer with fetal calf serum (FCS), and stored at 4°C in Stain Buffer with FCS (BD) for up to 8 hours, until flow cytometric analysis. Flow cytometry was performed using a 5-laser Cytek Aurora (Cytek Biosciences). Gating was performed using FlowJo (BD), and AIM^+^ gates were drawn based on MP-stimulated responses relative to DMSO responses. PBMC quality was evaluated by measuring the median response to SEB for all samples. PBMC samples with responses less than 50% of the overall median SEB response were excluded from downstream analyses.

SI for each sample was calculated by the fold change in the AIM^+^ response in the MP-stimulated condition compared with the average DMSO response for the same sample. An SI cutoff of 2 was applied for AIM^+^CD4^+^ T cell responses, and samples that failed to demonstrate at least a 2-fold response above background (by SI) were excluded from analysis of background-subtracted AIM responses. An SI cutoff of 3 was used for AIM^+^CD8^+^ T cell responses. Background-subtracted AIM^+^ responses were calculated for samples not excluded by the SI criteria by subtracting the DMSO background from antigen-specific (spike or non-spike) T cell responses, with a minimal DMSO level set to 0.001% (44). The LOQ was calculated using the geometric mean of all DMSO wells multiplied by the geometric SD factor. Positive responders were defined by those who had background-subtracted responses greater than the LOQ. All nonresponder values (background-subtracted AIM^+^ response < LOQ) were set at baseline (0.5 × LOQ).

### Hybrid AIM+ICS assay.

PBMCs were thawed and plated in parallel and as described above for AIM assays. Notably, the CD8-RE MP was also used for AIM+ICS. No chemokine receptor antibodies were added on day 1 for the AIM+ICS assays (see Supplemental Table 1 for antibodies used for AIM+ICS). After 20 to 22 hours, PMA (0.05 μg/mL) and ionomycin (0.25 μg/mL) were added to the positive (ICS) control wells. Two hours later, 0.25 μL/well of GolgiStop (BD) and GolgiPlug (BD) and the AIM marker Ab (Supplemental Table 1) were added to all samples, and the plates were incubated for another 4 hours at 37°C (in a 5% CO_2_ incubator). Cells were then washed, surface stained for 30 minutes at 4°C, fixed, and washed using Cytofix/Cytoperm (BD) per the manufacturer’s protocol. ICS was then performed using antibodies diluted in Perm/Wash Buffer (BD) for 30 minutes at 4°C. Cells were washed with Stain Buffer with FCS and stored in this buffer at 4°C until flow cytometric analysis was performed using a Cytek Aurora. For AIM+ICS the minimal DMSO level was set to 0.005% for background subtraction. Gating and SI calculations were performed as described above for AIM. CD4^+^ T cell responses with SI < 2 and CD8^+^ T cell responses with SI < 3 were excluded from downstream analyses, as described above for AIM. The LOQ was set at 0.01 for background-subtracted CD8^+^ T cell responses. Otherwise, LOQ was calculated as described above for AIM.

### Serology.

Serum binding antibody assays were performed to evaluate IgG responses to SARS-CoV-2 nucleocapsid and ancestral spike S2 domains and RBD using the Bio-Plex Pro Human SARS-CoV-2 IgG (N, S2, RBD) 4-Plex Panel serology assay (Bio-Rad 12014634) per the manufacturer’s protocols. This assay uses the MFI of a serological control (VIROTROL SARS-CoV-2, Bio-Rad) to generate a standard curve, which can be used to calculate semiquantitative IgG titers in AU/mL. Data are shown for samples with titers that fell within the working range for the standard curve. All samples with titers that fell outside of the working range for the standard curve were excluded from analyses. IgG titers were also converted to binding antibody units/mL using the manufacturer’s recommended conversion factors for converting Bio-Plex Pro IgG titers in AU/mL to the World Health Organization NIBSC 20/136 standard IgG titers in binding antibody units/mL. The conversion factors were 0.0008 for N, 0.0007 for S2, and 0.0027 for RBD IgG. The lower limit of quantification and MFI-based cutoffs for positivity for serology assays are indicated in the figures as per the figure legends. Please see individual figures for additional details.

### Data sharing.

The authors confirm that the data underlying the findings are fully available. Due to ethical restrictions, additional ACTIV-2/A5401 clinical trial study data beyond what are presented in this manuscript and supplement are available upon request from sdac.data@sdac.harvard.edu with the written agreement of the AIDS Clinical Trials Group and the manufacturer of the investigational product.

### Statistics.

Statistical analyses were performed in GraphPad Prism 9 (GraphPad Software). Fisher’s exact tests were used to compare T cell response rates to SARS-CoV-2 MPs between treatment and control groups. Mann-Whitney nonparametric tests were used for comparisons between the treatment and placebo groups for each stimulation condition(s) and/or cell type(s) between equivalent mAb treatment and placebo group conditions. Kruskal-Wallis nonparametric tests with post hoc Dunn’s multiple-comparison tests were used for assessing T cell responses across more than 1 stimulation condition or cell type. Comparisons of antibody titers between mAb treatment and placebo groups were also determined by Mann-Whitney tests. For all analyses a 2-sided *P* value of less than 0.05 was considered significant. Additional details of analyses are as described in the corresponding results sections and figure legends.

### Study approval.

The ACTIV-2/A5401 clinical trial protocol was approved by a central institutional review board (IRB): Advarra (Pro00045266, Columbia, Maryland, USA). The La Jolla Institute for Immunology IRB provided additional review and approval of this study. All participants enrolled in ACTIV-2/A5401 provided written informed consent for participation. The trial is registered at ClinicalTrials.gov Identifier: NCT04518410.

## Author contributions

SIR, DMS, and SC conceptualized the study; SIR, AH, and FF investigated; SIR and SC performed formal analysis for the study; DMS recruited patients and provided samples; DMS, KWC, MDH, JJE, JSC, DAW, ESD, JR, and CM were involved in clinical trials design and oversight; UMP, SFS, and AH provided data and resources for serologic analyses; AG, DW, PK, and AS provided additional material resources; SIR, FF, PK, DMS, and SC wrote the manuscript; and DMS and SC supervised the study.

## Supplementary Material

Supplemental data

## Figures and Tables

**Table 1:**
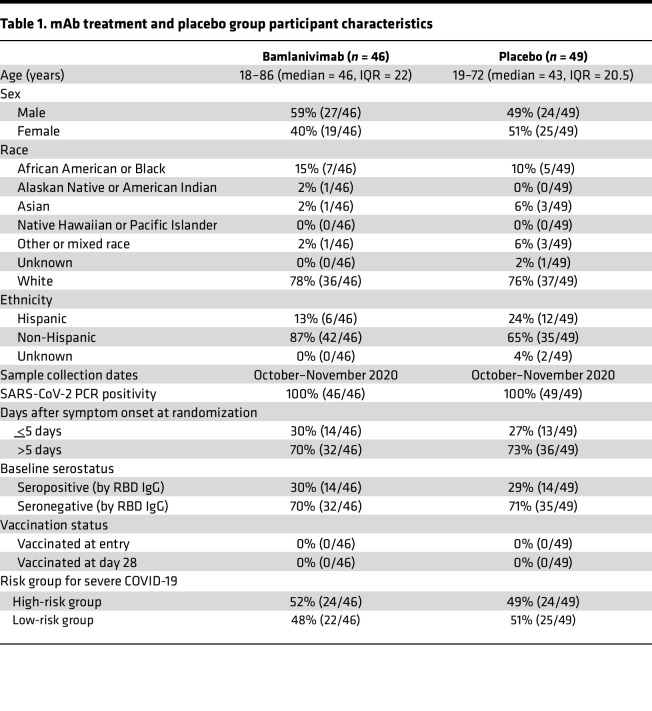
mAb treatment and placebo group participant characteristics

## Version 1 11/15/2022

In-Press Preview

## Version 2 12/22/2022

Electronic publication
