# Supplementary material for: Bamlanivimab therapy for acute COVID-19 does not blunt SARS-CoV-2–specific memory T cell responses
Source: JCI Insight. 2022 Dec 22;7(24):e163471. doi: 10.1172/jci.insight.163471 (PMC9869965; doi:10.1172/jci.insight.163471)
Supplement: Supplemental data [file jciinsight-7-163471-s020.pdf]

SUPPLEMENTAL FIGURES

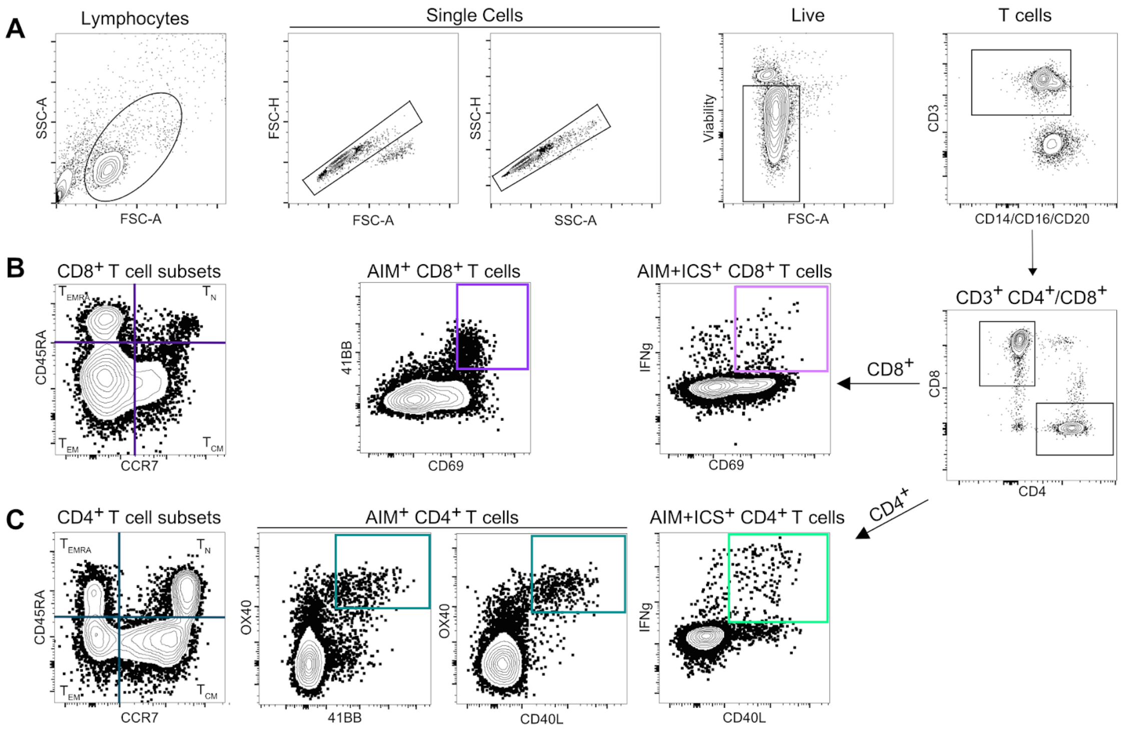

Figure S1. Gating for identification of SARS-CoV-2-specific CD4⁺ and CD8⁺ T cells; related to Figures 1-6 and Supplemental Figures 2-6. A. Gating strategy for identification of SARS-CoV-2-antigen-specific T cells. B. Gating strategy for identification of SARS-CoV-2-antigen-specific CD8⁺ T cells by subtype (left), AIM (center), AIM+ICS (right). C. Gating strategy for identification of SARS-CoV-2-antigen-specific CD4⁺ T cells by subtype (left), AIM (center), AIM+ICS (right).

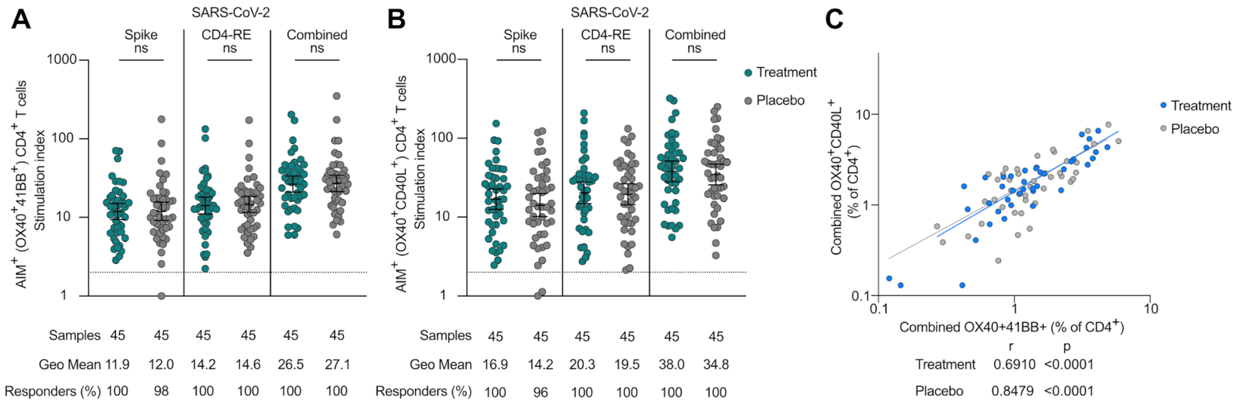

Figure S2. **SARS-CoV-2-specific CD4⁺ T cell responses; related to Figure 1.**
A. Stimulation index (fold-change above background; minimum value of 1) for spike, CD4-RE, or combined SAR-CoV-2-specific CD4⁺ T cells (surface OX40⁺41BB⁺CD4⁺ T cells) at study day 28 in individuals who received mAb (Treatment; teal circles) or placebo control (gray circles) for acute COVID-19 by AIM assay following 24-hour stimulation of PBMC with SARS-CoV-2 spike or CD4-RE peptide megapools (MPs). Combined AIM assay CD4⁺ T cell responses were calculated as the sum of the responses to individual (spike and CD4-RE) MPs. The dotted black line indicates the LOQ (equal to 2). Error bars represent geometric mean with geometric standard deviation; ns equals not significant. B. Stimulation index for spike, CD4-RE, or combined SAR-CoV-2-specific CD4⁺ T cells as in A but using surface OX40⁺CD40L⁺CD4⁺ T cells to identify AIM⁺ CD4⁺ T cells. C. Correlation analysis of SARS-CoV-2-specific CD4⁺ T cells by AIM assay using the AIM surface markers combinations of OX40⁺41BB⁺ and OX40⁺CD40L⁺. The percentage of total AIM⁺ SARS-CoV-2-specific OX40⁺41BB⁺CD4⁺ and OX40⁺CD40L⁺CD4⁺ T cells at study day 28 in individuals who received mAb (Treatment; blue circles and blue trendline) or placebo control (gray circles and gray trendline) for acute COVID-19 following 24-hour stimulation with SARS-CoV-2 MPs were plotted; Spearman r and p values for Treatment and Placebo groups are shown.

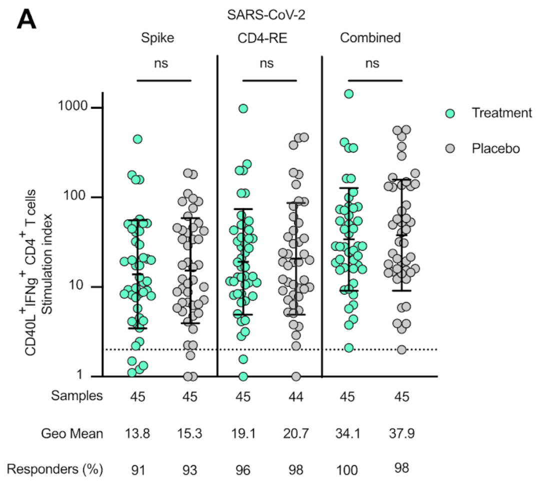

Figure S3. SARS-CoV-2-specific CD4⁺ T cell functionality; related to Figure 2.

A. Stimulation index (fold-change above background; minimum value 1) for spike, CD4-RE, or combined SAR-CoV-2-specific $CD4^+$ T cells (surface $CD40L^+$ intracellular $IFNg^+CD4^+$ T cells) at study day 28 in individuals who received mAb (Treatment; light green circles) or placebo control (gray circles) for acute COVID-19 by hybrid AIM + ICS assay following 24-hour stimulation of PBMC with SARS-CoV-2 spike or CD4-RE peptide megapools. Combined AIM + ICS assay $CD4^+$ T cell responses were calculated as the sum of the responses to individual (spike and CD4-RE) peptide megapools. The dotted black line indicates the LOQ (equals 2). Baseline set at 1. Error bars represent geometric mean with geometric standard deviation. Pairwise comparisons were made between equivalent stimulation conditions for Treatment and Placebo groups by Mann-Whitney nonparametric statistical testing; 'ns' equals not significant.

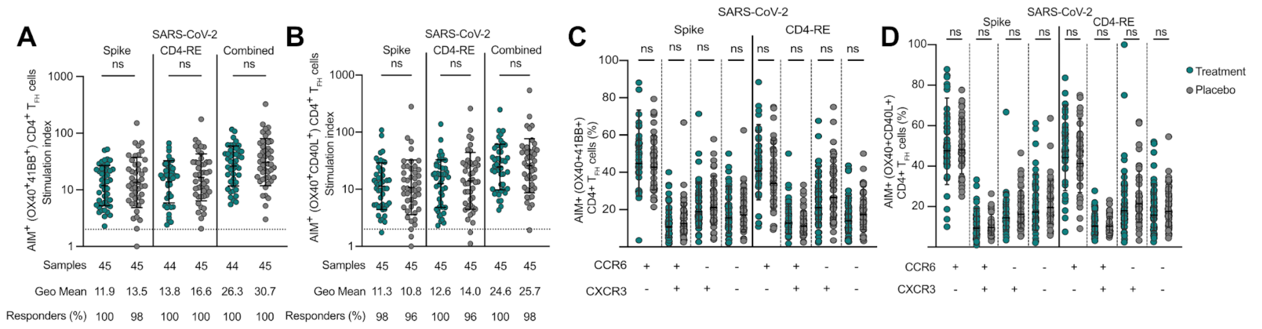

Figure S4. SARS-CoV-2-specific T_{FH} populations; related to Figure 3.

A, B. Stimulation index (fold-change above background; minimum value 1) for spike, CD4-RE, or combined SAR-CoV-2-specific T_{FH} cells (surface OX40$^+$41BB$^+$CXCR5$^+$CD4$^+$ T_{FH} cells in A and surface OX40$^+$CD40L$^+$CXCR5$^+$CD4$^+$ T_{FH} cells in B) at study day 28 in individuals who received mAb (Treatment; teal circles) or placebo control (gray circles) for acute COVID-19 by AIM assay following 24-hour stimulation of PBMC with SARS-CoV-2 spike or CD4-RE peptide megapools. Combined AIM assay CD4$^+$ T cell responses were calculated as the sum of the responses to individual (spike and CD4-RE) peptide megapools. The dotted black line indicates the LOQ (equals 2). Baseline set at 1. Error bars represent geometric mean with geometric standard deviation; ns equals not significant. C, D. Percentage of spike, CD4-RE, or combined SAR-CoV-2-specific circulating T follicular helper T cells (T_{FH}; CXCR5$^+$ as percentage of AIM$^+$ CD4$^+$ T cells) expressing surface CXCR3 and/or CCR6 at study day 28 in individuals who received mAb (Treatment; teal circles) or placebo control (gray circles) on study entry for acute COVID-19 by AIM assay following 24-hour stimulation of PBMC with SARS-CoV-2 spike or CD4-RE MPs; AIM markers included surface OX40 and 41BB (C) or OX40 and CD40L (D). Combined T_{FH} responses were calculated as the sum of the T_{FH} specific to the individual (spike and CD4-RE) MPs. Bars represent geometric mean with geometric standard deviation. Pairwise testing by Mann-Whitney; ns equals not significant.

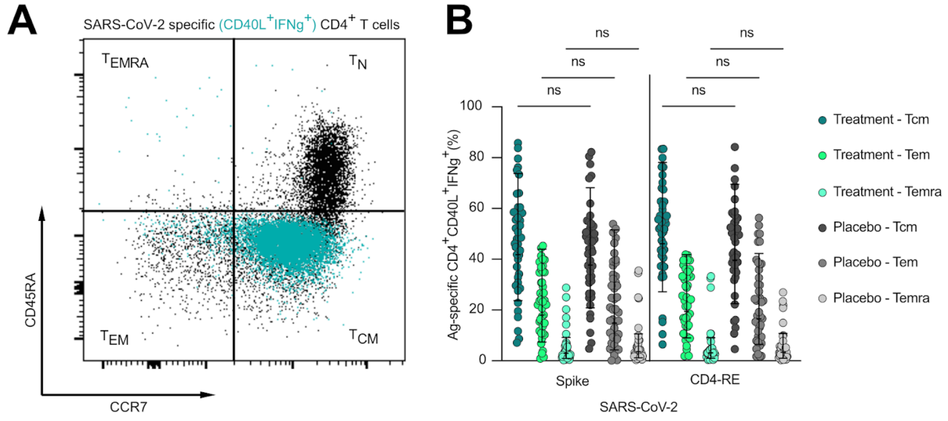

Figure S5. SARS-CoV-2-specific CD4+ memory T cell subsets are equivalent following receipt of mAb or placebo; related to Figure 4. A. Representative flow cytometry plots of total circulating naïve and memory CD4+ T cell subsets (black) and proportion of naïve/memory cells that are SARS-CoV-2-specific CD4+ T cells by AIM+ICS (teal overlay; CD40L+IFNg+). B. Percentage of SARS-CoV-2-specific CD4+ T cells that are T central memory (Tcm), T effector memory (Tem) and terminal effector cells (Temra) at study day 28 in individuals who received mAb (Treatment; 3 shades of teal/green circles) or placebo control (3 shades of gray circles) for acute COVID-19 by AIM+ICS (CD40L+IFNg+) following 24-hour stimulation with SARS-CoV-2 spike or CD4-RE peptide megapools. T cell memory subtype (Tcm, Tem, Temra) was assigned based on surface expression of CCR7 and/or CD45RA. Error bars represent geometric mean with geometric standard deviation. Equivalent memory T cell populations for Treatment and Placebo groups were compared by Kruskal-Wallis tests with Dunn's post-hoc correction for multiple comparisons; ns equals not significant.

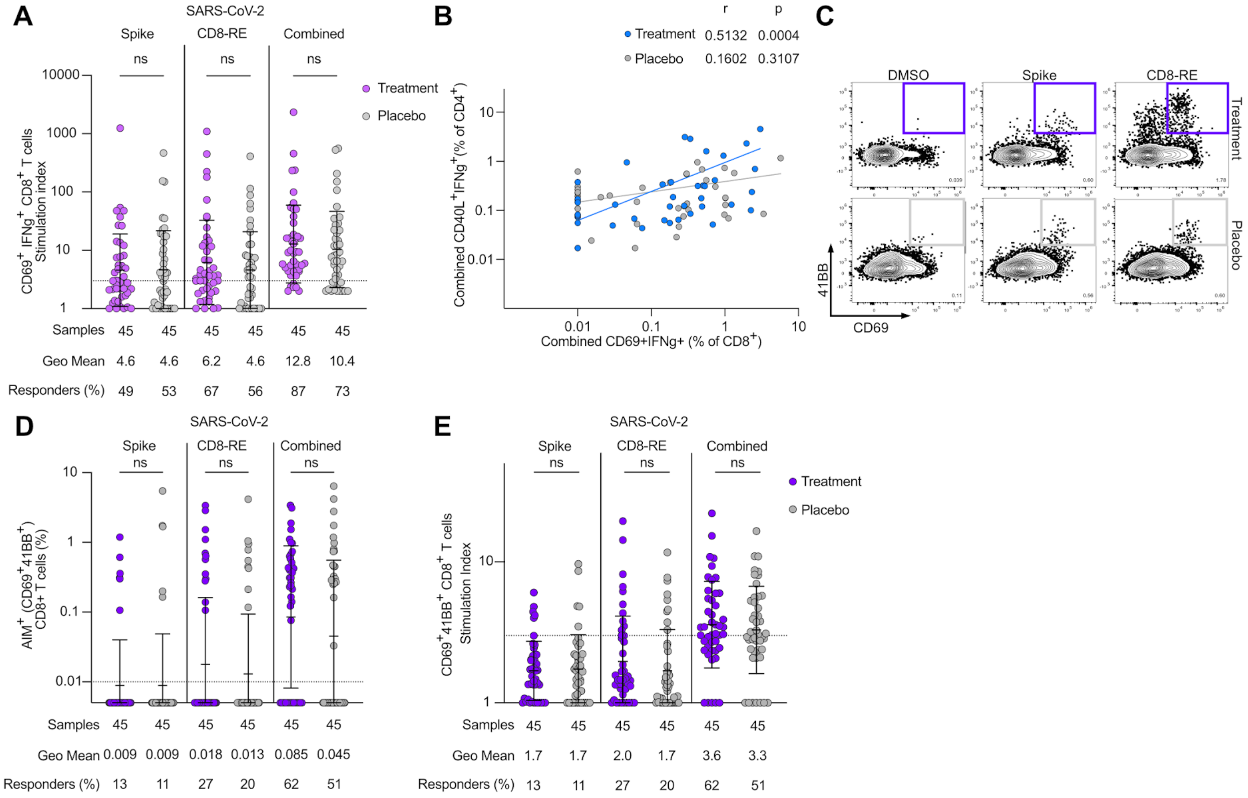

Figure S6. SARS-CoV-2-specific CD8⁺ T cell responses and functionality; related to Figure 5 and 6.

A. Stimulation index (SI) (fold-change above background; minimum value 1) for spike, CD8-RE, or combined SAR-CoV-2-specific CD8⁺ T cells (surface CD69⁺ intracellular IFNg⁺CD8⁺) by hybrid AIM + ICS following 24-hour stimulation of PBMC with SARS-CoV-2 spike or CD8-RE peptide megapools (MPs) at study day 28 in individuals who received mAb (Treatment; lavender circles) or placebo (gray circles) for acute COVID-19. Combined responses equal the sum of responses to the individual MPs. Dotted black line indicates LOQ (equals 3). Error bars represent geometric mean with geometric standard deviation; ns equals not significant. B. Correlation between SARS-CoV-2-specific CD4⁺ and CD8⁺ T cells by AIM + ICS at day 28 using surface CD40L⁺or CD69⁺ and intracellular IFNg⁺, respectively, in mAb (Treatment; blue circles with blue trendline) and Placebo (gray circles with gray trendline) groups; Spearman r and p values are shown. C. Representative flow cytometry plots of SARS-CoV-2-specific CD8⁺ T cells at study day 28 in mAb (Treatment; upper, purple boxes) or Placebo (lower, gray boxes) groups using the AIM markers CD69⁺41BB⁺ (see Fig. S1 for ancestral gating). D. Background subtracted spike, CD8-RE, or combined SARS-CoV-2-specific CD8⁺ T cells by AIM (surface CD69⁺41BB⁺) as a percentage of CD8⁺ T cells at study day 28 in mAb (Treatment; purple circles) and Placebo (gray circles) groups following 24-hour stimulation of PBMC with SARS-CoV-2 spike and/or CD8-RE MPs. Combined responses equal the sum of responses to individual MPs. Dotted black line indicates LOQ (equals 0.01). Baseline set at 0.5 LOQ. Values for all non-responders (SI <3 in Fig. S6E) were set to baseline. Error bars represent geometric mean with geometric standard deviation; ns equals not significant. E. AIM⁺ SARS-CoV-2-specific CD8⁺ T cells as in D but by SI. Dotted black line (LOQ) equals SI of 3. Baseline set at 1.

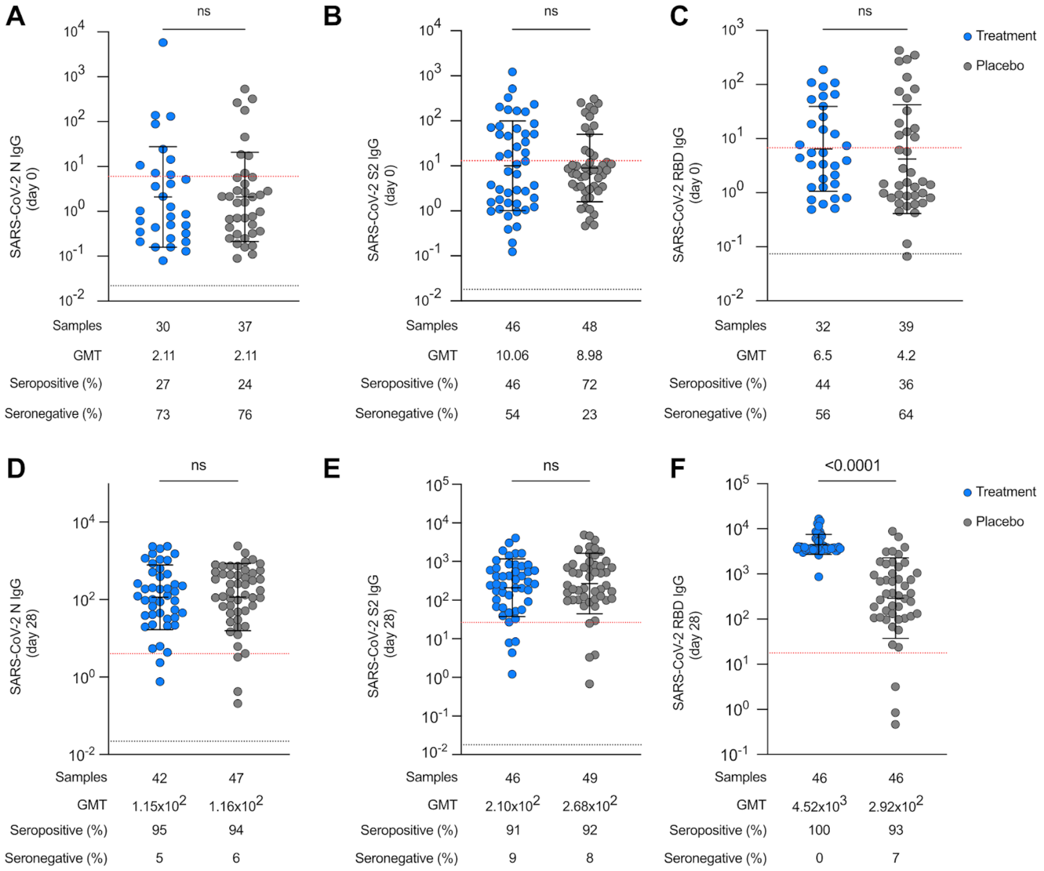

Figure S7. SARS-CoV-2-specific baseline and day 28 IgG titers in BAU/mL; related to Figure 7.
All SARS-CoV-2 IgG titers generated by Bio-Plex assay and expressed as AU/mL in Fig. 7 were converted based on the WHO standard NIBSC 20/136 and expressed as BAU/mL here. A-C. Baseline IgG titers for ancestral SARS-CoV-2 N (in A), S2 (in B), and RBD (in C) at study entry (day 0) in individuals who received bamlanivimab (Treatment; blue circles) versus placebo (Placebo; gray circles). D-F. Day 28 IgG titers for ancestral SARS-CoV-2 N (in D), S2 (in E), and RBD (in F) in Treatment (blue circles) and Placebo (gray circles) groups. Dotted black line indicates lower limit of quantification of detection (titer) for Bio-Plex (N, S2, RBD) serologic assays in BAU/mL. Dotted red line indicates cutoff for MFI-based positivity for BioPlex assays in BAU/mL; titer results were generated using a standard curve generated by standards provided by the manufacturer [out of range titers were excluded from analyses] and then converted to BAU/mL using the conversion factors provided by the manufacturer. MFI-based positivity was set based on results generated using pre-pandemic, SARS-CoV-2 uninfected control samples, which were then converted to BAU/mL.

SUPPLEMENTAL TABLES

Table S1. Antibodies

Marker-Fluorophore	Clone	Vendor	Catalog #	Assay
Fixable live/dead blue	N/A	Thermo Fisher	L34962	AIM, AIM+ICS
CD3-BUV395	UCHT1	BD	563546	AIM, AIM+ICS
CD4-cFluor B548	SK3	Cytek	SKU R7-20044	AIM, AIM+ICS
CD8a-BUV805	SK1	BD	612889	AIM, AIM+ICS
CD14/CD16/CD20-BV510	63D3/3G8/2H7	BioLegend	367123/302048/302340	AIM, AIM+ICS
CD45RA- BV570	HI100	BioLegend	304132	AIM, AIM+ICS
CCR7-BV711	G043H7	BioLegend	353228	AIM
CCR7-PE-Cy7	G043H7	BioLegend	353226	AIM+ICS
OX40-APC	Ber-Act35	BioLegend	350008	AIM
OX40-APC/Fire750	Ber-Act35	BioLegend	350031	AIM+ICS
CD137-BUV737	4B4-1	BD	568348	AIM
CD137-PE-Cy5	4B4-1	BioLegend	309808	AIM+ICS
CD40L-PE/Dazzle594	24-31	Biolegend	310840	AIM
CD40L-PerCP-eF710	24-31	Thermo	46-1548-42	AIM+ICS
CD25-APC/Fire750	BC96	BioLegend	302642	AIM
CD25-BV650	BC96	BioLegend	302634	AIM+ICS
CD69-FITC	FN50	BioLegend	310904	AIM
CD69-BV605	FN50	BioLegend	310938	AIM+ICS
CCR6-BUV496	11A9	BD	612948	AIM
ICOS-BUV563	DX29	BD	741421	AIM
CXCR5-BV421	J252D4	BioLegend	356920	AIM
CXCR3-BV605	G025H7	BioLegend	353728	AIM
CD38-BV650	HB-7	BioLegend	356620	AIM
PD-1-BV785	EH12.2H7	BioLegend	329930	AIM
CD95	DX2	BD	566542	AIM
PD-L1-PE	29E.2A3	BioLegend	329706	AIM
HLA-DR-APC-R700	Tu39	BD	746979	AIM
CCR4-PE-Cy7	L291H4	BioLegend	359410	AIM+ICS
IFNγ-AF488	4S.B3	BioLegend	502515	AIM+ICS
IL-2-BB700	MQ1-17H12	BD	566405	AIM+ICS
IL-4-BUV737	MP4-25D2	BD	612835	AIM+ICS
IL-10-PE/Dazzle594	JES3-19F1	BioLegend	506812	AIM+ICS
IL-17a-BV785	BL168	BioLegend	512338	AIM+ICS
IL-21-PE	4BG1	BioLegend	516704	AIM+ICS
GzmB-AF647	GB11	Biolegend	515406	AIM+ICS
TNFα-eF450	Mab11	eBioscience	48-7349-42	AIM+ICS

SUPPLEMENTAL ACKNOWLEDGMENTS

ACTIV-2/A5401 Study Team

Kara Chew, MD, MS, Co-Chair, David Geffen School of Medicine at University of California, Los Angeles, Los Angeles, CA, USA

David (Davey) Smith, MD, MAS, Co-Chair, University of California, San Diego, La Jolla, CA, USA

Eric Daar, MD, Vice Chair, Lundquist Institute at Harbor-UCLA Medical Center, Torrance, CA, USA

David Wohl, MD, Vice Chair, University of North Carolina at Chapel Hill School of Medicine, Chapel Hill NC, USA

Judith Currier, MD, MSc, Protocol Investigator and ACTG Chair, David Geffen School of Medicine at University of California, Los Angeles, Los Angeles, CA, USA

Joseph Eron, MD, Protocol Investigator and ACTG Vice Chair, University of North Carolina at Chapel Hill School of Medicine, Chapel Hill NC, USA

Arzhang Cyrus Javan, MD, MPH, DTM&H, NIH Division of AIDS (DAIDS) Clinical Representative, National Institutes of Health, Rockville, MD, USA

Michael Hughes, PhD, Lead Statistician, Harvard T.H. Chan School of Public Health, Boston, MA, USA

Carlee Moser, PhD, Statistician, Harvard T.H. Chan School of Public Health, Boston, MA, USA

Mark Giganti, PhD, Statistician, Harvard T.H. Chan School of Public Health, Boston, MA, USA

Justin Ritz, MS, Statistician, Harvard T.H. Chan School of Public Health, Boston, MA, USA

Lara Hosey, MA, Clinical Trials Specialist, AIDS Clinical Trials Group (ACTG) Network Coordinating Center, Social & Scientific Systems, a DLH Company, Silver Spring, MD, USA

Jhoanna Roa, MD, Clinical Trials Specialist, AIDS Clinical Trials Group (ACTG) Network Coordinating Center, Social & Scientific Systems, a DLH Company, Silver Spring, MD, USA

Nilam Patel, Clinical Trials Specialist, AIDS Clinical Trials Group (ACTG) Network Coordinating Center, Social & Scientific Systems, a DLH Company, Silver Spring, MD, USA

Kelly Colsh, PharmD, DAIDS Pharmacist, NIH/DAIDS Pharmaceutical Affairs Branch, Rockville, MD, USA

Irene Rwakazina, PharmD, DAIDS Pharmacist, NIH/DAIDS Pharmaceutical Affairs Branch, Rockville, MD, USA

Justine Beck, PharmD, DAIDS Pharmacist, NIH/DAIDS Pharmaceutical Affairs Branch, Rockville, MD, USA

Scott Sieg, PhD, Protocol Immunologist, Case Western Reserve University, Cleveland, OH, USA

Jonathan Li, MD, MMSc, Protocol Virologist, Brigham and Women's Hospital, Harvard Medical School, Boston, MA, USA

Courtney Fletcher, PharmD, Protocol Pharmacologist, University of Nebraska Medical Center, Omaha, NE, USA

William Fischer MD, Protocol Critical Care Specialist, University of North Carolina at Chapel Hill School of Medicine, Chapel Hill NC, USA

Teresa Evering, MD, MS, Protocol Investigator, Weill Cornell Medicine, New York, NY, USA

Rachel Bender Ignacio, MD, MPH, Protocol Investigator, University of Washington, Seattle, WA, USA

Sandra Cardoso, MD, PhD, Protocol Investigator, Fundação Oswaldo Cruz, Rio de Janeiro, Brazil

Katya Corado, MD, Lundquist Institute at Harbor-UCLA Medical Center, Torrance, CA, USA

Prasanna Jagannathan, MD, Protocol Investigator, Stanford University School of Medicine, Palo Alto, CA, USA

Nikolaus Jilg, MD, PhD, Protocol Investigator, Massachusetts General Hospital, Harvard Medical School, Boston, MA, USA

Alan Perelson, PhD, Protocol Investigator, Los Alamos National Laboratory, Los Alamos, NM, USA

Sandy Pillay, MB, CHB, Protocol Investigator, Enhancing Care Foundation, Durban, KwaZulu-Natal, South Africa

Cynthia Riviere, MD, Protocol Investigator, GHESKIO Center, Port-au-Prince, Haiti

Upinder Singh, MD, Protocol Investigator, Stanford University School of Medicine, Palo Alto, CA, USA

Babafemi Taiwo, MBBS, MD, Protocol Investigator, Northwestern University Feinberg School of Medicine, Chicago, IL, USA

Joan Gottesman, BSN, RN, CCRP, Field Representative, Vanderbilt University Medical Center, Nashville, TN, USA

Matthew Newell, BSN, RN, CCRN, Field Representative, University of North Carolina at Chapel Hill School of Medicine, Chapel Hill NC, USA

Susan Pedersen, BSN, RN, Field Representative, University of North Carolina at Chapel Hill School of Medicine, Chapel Hill NC, USA

Joan Dragavon, MLM, Laboratory Technologist, University of Washington, Seattle, WA, USA

Cheryl Jennings, BS, Laboratory Technologist, Northwestern University, Chicago, IL, USA

Brian Greenfelder, BA, Laboratory Technologist, Ohio State University, Columbus, OH, USA

William Murtaugh, MPH, Laboratory Specialist, ACTG Laboratory Center, University of California, Los Angeles, Los Angeles, CA, USA

Jan Kosmyna, MIS, RN, CCPR, ACTG Community Scientific Subcommittee Representative, Case Western University Clinical Research Site, North Royalton, OH, USA

Morgan Gapara, MPH, International Site Specialist, ACTG Network Coordinating Center, Social & Scientific Systems, a DLH Company, Durham, NC, USA

Akbar Shahkolahi, PhD, International Site Specialist, ACTG Network Coordinating Center, Social & Scientific Systems, a DLH Company, Silver Spring, MD, USA

Paul Klekotka, MD, PhD, Industry Representative, Lilly Research Laboratories, San Diego, CA, USA
